# Normality in medicine: a critical review

**DOI:** 10.1186/s13010-020-00087-2

**Published:** 2020-04-16

**Authors:** Marisa Catita, Artur Águas, Pedro Morgado

**Affiliations:** 1 Life and Health Sciences Research Institute (ICVS), School of Medicine, University of Minho, Campus de Gualtar, 4710-057 Braga, Portugal; 2 ICBAS – Instituto de Ciências Biomédicas de Abel Salazar, Universidade do Porto, Porto, Portugal; 3 ICVS-3Bs PT Government Associate Laboratory, Braga/Guimarães, Portugal

**Keywords:** Normality, Medical ethics, Cultural medicine, Psychiatry

## Abstract

What is considered normal determines clinical practice in medicine and has implications at an individual level, doctor-patient relationship and health care policies. With the increase in medical information and technical abilities it is urgent to have a clear concept of normality in medicine so that crucial discussions can be held with unequivocal terms.

The different meanings for normality were analyzed throughout the literature and grouped according to their relevance in the academic community in models, namely the Biostatistical Theory (BST), Health, Ideal, Process and Biological advantage. The BST is the most established naturalistic approach, however normal variability can arguably constitute a problem. Health is similar and raises the question of setting the boundaries of pathology. Normality as an Ideal is an useful tool but is naturally unrealistic. As a Process it is comprehensible but is hard to frame for practical purposes. If considered as a Biological Advantage, seems intuitive but abnormality should tend to disappear.

After, three examples were presented to discuss these models. They were Anemia, Psychiatric diseases and Psychopathy. In the case of Anemia the BST was applied and the arbitrary boundaries but with social impact were exposed. Psychiatric diseases was discussed under the process of self-organization and non-suffering ideal. With Psychopathy the boundaries of biological advantage are questioned.

This review appeals to the importance of redesigning of the concept of normality in medicine according to current times and stresses the importance of integrating concepts such as variability and autonomy.

## Abreviations

BST Biostatistical Theory.

DSM Diagnostic and Statistical Manual of Mental Disorders.

Hb blood hemoglobin concentration.

Hct hematocrit.

HDA Harmful dysfunction analysis.

HST Holistic Theory of Health.

WHO World Health Organization.

## Introduction

In clinical practice normality is at the basis of all comparisons. [7] From normality derives both health and disease, with implications from the patient self-perception and doctor-patient communication, to the goal of medical intervention, health insurance policies and public health measures [2, 8, 9, 14]. The proliferation of medical information, which is at the general public disposal, and the technological possibilities should keep pace with conceptual and ethical literacy. Alarming interpretations proliferate to the detriment of a balanced relationship with health issues. Diagnostics and therapeutics to achieve a given normality cause public instability worth attention, since this changing nature of health is unavoidable and normalizing parameters is not a solution to a balanced approach to life. Since there are tools to alter conditions the main focus should be in defining when to intervene.

Medicine is said to be the most humane of sciences and the most scientific of humanities. As a science striving for its objectivity and as a humanity in search of understanding, the concepts that lay the foundation for medicine must be clearly defined [2]. With the variety of subjects where the term normality is applied, and with different meanings, misunderstandings occur easily.

Philosophers of medicine regard normality either directly or indirectly along with the concept of health and disease. In the first place, the foundations for the theory of medicine must be considered.

Historically, Plato and Aristotle convey that medicine produces health. Plato understood health in a hierarchical order, whereby health would mean the supremacy of the soul over the body, and thus the rational part would surpass the desirous one [10]. From Aristotle’s view that motion is an actualization of a potentiality, health can be interpreted as the ability to accomplish goals, being healthier the one with more options or possibilities [10].

Modern medicine arises in the nineteenth century, and according to Canguilhem and Foulcaut point of view, it is a product of two revolutionary changes in the clinical orientation that organized the theory of medicine into ontological and physiological [3]. The ontological was the endeavor to correlate anatomic pathology to clinical signs and symptoms. Then physiology brought an experimentally-based medicine, as by the 1820s Broussais observed that the normal and pathological could be a matter of different intensity, in a quantifiable function with continuous values [3, 9, 14]. Pasteur in the mid nineteenth century with his experimental work on the germ theory fits the ontological conception, whereas in the 20 century the French and German reductionist schools were prone to quantify physiological processes [14].

Also worth noticing is the impact from the model of biological order and function that Darwin brought with the publication On the Origin of Species, postulating an ever-changing species which can be extrapolated to such subjects as immunology [14].

Among the most prominent theorists in contemporary philosophy of medicine are Christopher Boorse for his Biostatistical Theory of Health, and Georges Canguilhem who is frequently cited for his conception of health as a dynamic state of changing norms.

The literature on the conceptualization of normality in medicine was analyzed through this revision with a view to better understand the models suggested thus far, what are their advantages and disadvantages in terms of how they fit current usage and how they promote adequate communication and subsequent decisions. No patients were involved in this work.

### Normality models

Normality has no consensual definition in medical literature. Not only the meaning varies, but also does the way it is conceptualized. In a simplistic view, it can have a naturalistic or normative approach [9]. The former tries to identify what the term means for the ones who apply it, independent of value judgments. The normative as a more constructive intention, where the meaning is established by the theorists [6].

From the articles studied were chosen the models that stood out as more relevant, for the strength of their arguments and their popularity amidst scholars. They constitute oversimplifications to help clarify each abstract rationalization and along this path they sometimes overlap each other.

#### Biostatistical theory (BST)

The BST of health, is a well-established naturalistic analysis by Christopher Boorse, discussed and revised since the seventies [2]. According to it health is synonym of normality, and represents the total absence of pathological conditions [2].

In brief, health is normal functional ability for a member of the reference class. This normal function refers to a statistically typical contribution by a part or process to their individual survival [or] reproduction. The reference class is determined by the age group and sex, translating an uniform functional design. A pathological condition is then the internal state in which these normal functions are impaired below the typical efficiency [2].

This theory unifies multiple concepts, some of them discussed below, and is adequate for many practical contexts, being its main advantages its naturalistic and quantifiable approach, both important to attain objectivity and fairness. Since this theory is comprehensive and the author did several publications and adaptations in rebuttal to many of the critics, it is cherished for its cohesion and for allowing vigorous debate [2].

On the other hand, the simple fact that this definition would mean that an individual is only normal when healthy and that an individual with any disease is abnormal is something to bear in mind regarding its implications for the societal views on disease.

As Koeslag points out, normal is associated with usual [7]. This means that no matter what function is measured, the same proportion of asymptomatic individuals would be considered outside the range of normal (5% is the value used by default), and the subnormal tail considered diseased by Boorse. With 10 independent tests for different functions, the probability of having at least one extra-normal value is of 42%, whereas if 25 tests are done, the chance is of 75%. Inversely, all diseases would have the same frequency [7]. These are of course major limitations to the use of a biostatistical approach since it has no good integration of variation and simple polymorphism.

#### Health

Normality understood as health is a prevalent view in medical philosophy. The terms are used interchangeably [7]. Different philosophers give some nuances to the conception of health, which can be either classified as naturalistic or normative.

Nordenfelt presents a definition for health in his Holistic Theory of Health (HST), that identifies health with the ability to reach vital goals in standard circumstances [13]. Wakefield presents the harmful dysfunction analysis (HDA), adding that part-dysfunction is not sufficient for considering a given condition a disease [15].

On a social scale defining the expectations of what is normal determines, for example, insurance coverage [14]. Even if health has not a clear definition, the promotion of health is generally seen as the goal of clinical medicine [13].

According to Wakefield, with the HDA, being value laden is necessary for analyzing the foundations of a practical activity like medicine versus a pure science, therefore this is an sine qua non condition for any conception of normality as health [15]. This is in conflict with the naturalistic approach suggested by Boorse [2].

Nordenfelt defends that health is not normality inasmuch conditions are not diseases because they are abnormal. Cancer is not a disease because it is abnormal but because it entails physiological dysfunctions [13].

Noting that incidental cases of flu are normal, but not healthy, health can be regarded as a special case of normality [9]. This leads to the preeminent challenge of demarcating between the normal and the pathological.

#### Ideal

Vesalius depicted an anatomy that no living human being has. The reductions that these depictions entail is of major didactic importance. Additionally, medical intervention entails objectives to be attained [9]. Simplifications are an essential undertaking when complex matters are operated since the reduction to categories allows actions to be taken [11]. It allows the formulation of guidelines, decision algorithms, and hence can also have legal functions. This is a naturalistic model of normality since determination of ideal proprieties is defined by optimal function and not by personal judgement.

On the other hand, this ideal attribute has no independent meaning. Nothing can be universally optimal. The best to a function might act in the detriment of other [7]. These are elusive expectations embedded in collective consciousness that are in truth unrealistic demands [14].

#### Process

According to Canguilhem, who contrasts between the normal and pathological instead of health and disease, normality is a process and reflects the ability to adapt to a certain context, internal and external [9]. The organism structures these environments, rather than submitting to them. There is self-organizatio n[14]. Individuals make their own decisions regarding what they consider best for their life [12]. Note that in this model normality is neither statistical nor the absence of disease [12].

Normality as a process has the advantage of integrating variability. Rudnick proposes that certain disabilities are so common in one’s life that they can be interpreted as normal variants [9]. These can be fully compensated by mechanisms of self-organization where alternatives are found to the disrupted structures and functions. From this perspective disability presents itself as pathological only when self-organization is impaired and there is a resulting handicap [9]. This view goes along with normality seen as the end of medical intervention since while medical technologies aim to prevent and treat diseases they also modify what is considered normal [12].

If normality is seen as a process than there is an inherent continuity associated with it. This implies drawing a line between health and disease that is inevitably somewhat arbitrary [11]. However, it cannot be reduced to an arbitrary subjective preference since the aimed outcome is the adaptation to the context [12]. Additionally, it can be harder to apply in legal contexts. Healthcare justice entails that disease inhibits equality of opportunity and that public health efforts should create mechanisms to protect it [11].

#### Biological advantage

Medicine and biology are affiliated, and their concepts intertwined. Darwin’s theory of natural selection is one of the most important concepts of biology and it states that inherited variations that increase the individual’s ability to compete, survive and reproduce are what drives evolution. This is a naturalistic approach that dismisses the formulation of a theoretical meaning. The analogy with normality in humans is that functions that don’t promote the purpose of evolution would be abnormal. Immunology is an example of the extension of this biological advantage model. In its principles the self is not a given entity, but rather it is a result of identifying the self and of the dynamic interaction with the external and adapts to it, being the best adaptation the one which strives [14].

This model key feature is it universality, since it applies the concept of normality to the human species as it would to any other [14]. Another aspect is the suitability to theories of the mind [14].

However if both the self and the environment are changing, so does the delineation of what constitutes a biological advantage [14]. If evolution is an ongoing process human functions would vary in their aim throughout history and normality could only be described linked to a period in time [7].

Another problem is that natural selection is a central mechanism in evolution and the best adaptation prevails, the analogy to health would mean that a reduction in pathology rates over time would be expected, even without medicine, which is not true [9].

### Examples

Bearing in mind these models the next section will comprise some examples, where the goal is to understand how certain entities can be analyzed in terms of normality.

#### Anemia

According to World Health Organization (WHO) criteria, anemia is defined as blood hemoglobin (Hb) concentration < 130 g/L (< 13 g/dL) or hematocrit (Hct) < 39% in adult males; Hb < 120 g/L (< 12 g/dL) or Hct < 37% in adult females. The signs and symptoms depend on the level and on the time course.

The demarcation here is objective and quantitative, the parameter varies only according to the sex. The BST seems a proper model to apply. The end point function is carrying oxygen throughout the body, this being essential to survival. Anemia is a priority worldwide, policies made, resources allocated, industries revolve over this subject and disinformation abounds. Moreover, by its 32.9% prevalence in general population, society as a whole is diseased to a great extent [1, 14]..

This definition of anemia is only quantitative and does not incorporate signs and symptoms which poses problems to the clinical value of the diagnosis. People with hemoglobin values outside this range can live without any other signs or symptoms, which exposes the problem of defining a diagnosis according to BST framework. The reasoning that explains how values outside this range determine loss of function, and how this lack of function (either in relation to reproduction or survival as the BST states) can be measured, seems to be the unanswered core question in this type of framework.

Interestingly, the Process framework would allow an approach that highlights changes during time in the same individual according to his/her clinical condition. More than the absolute value, the focus is on the interpretation of its variations.

#### Psychiatric diseases

The Diagnostic and Statistical Manual of Mental Disorders (DSM), is the American reference book for the diagnostic of mental illnesses, and its validity is continuously questioned. Whether mental illnesses are normal or not is a matter requiring serious analysis in a world where stigma and industry rule. The boundaries that determine whether someone is simply being human and complex or on the other hand alarmingly dysfunctional in need for serious therapy are blurry.

Obsessive-compulsive behaviors can be a motive for pride and success for some and a living nightmare for others. Their own perception of having a problem so often is the criteria for having it. Using the normality as Health framework, some of these repetitive behaviors could be seen as normal if not experienced as dysfunctional and/or inducing psychological suffering.

Discussed in the light of the Process framework, normality would be conceptually achieved tackling either the disfunction itself or the self-organization the subject has around it. In other words, this level of subjectivity suggests that either the doctor helps with changing the obsession, or, why not, rather changing the perception the patient has over his/her own life, full of patterns that are so particular to him that is for nobody to judge normal. It is hard (if not impossible) to make these diagnostics with no judgment values.

In the same vein, Fried and Agassi’s work on paranoia for example demarcate normal from the pathological in terms of a deficiency in cognitive self-correction, since the psychotic individual does not acknowledge a distinction between his distorted view and reality [5, 9]..

#### Psychopathy

Psychopathy, as in antisocial behavior and impaired empathy, remorse and egotistical traits can be nothing less than appropriate in a context where unfairness rules, where inequality is evident, and where suffering, if not seen before our eyes, is of well-known existence somewhere around the globe.

Wakefield sets his goal as distinguishing normal suffering from mental disorder, so that there is a solid foundation for critique of overly expansive psychiatric diagnosis that pathologizes normal variation [15]. Normality can be arguably seen as a process where there is adaptation to a certain context, with self-organization in terms of values, which can represent Biological Advantage, in the sense that it can be seen as a coping mechanism with harsh realities.

## Discussion

To meet the challenge of conceptualizing normality in medicine in such a way that it can be seen as a simple and practical tool, some of the points formerly discussed must be retained and others added.

To begin with, in the humble view of this analysis, normality in medicine cannot be a synonym with health. The arguments studied were not sufficiently strong to be worth the costs of such an assertion. The impact is so broad, it is both political and individual, and telling that the normality is health can only be appreciated as an ideal. Even when using statistical norm and subnormal values to evaluate the health of someone, using a naturalist approach, non-value laden, even if according to parameters used generally in biology, as in the most prevailing theory the BST, normality as health still implies a reduction in meaning. Even though the health care practitioner can normalize parameters, and to do so uses evidence-based medicine that applies statistics and ranges to the advantage of the patient morbidity and mortality rate, this is not enough to determine the best interest of a given individual. Being normal is more than experiencing health since it entails managing both healthy and non-healthy states. Likewise, unhealthy people could be considered normal when able to cope with disease. Self-limited events such as flu, gastroenteritis, anxiety or minor chronical conditions such as gastro-esophageal reflux disorder are frequent and can be understood in the light of this conception of normality.

This conception of normality suits the Biopsychosocial model contemplated by Engel as another element that articulates with its concepts of health, wellbeing, social well-being as well as disease, illness and sickness. According to the Biopsychosocial model, health has a biological, psychological and social dimension that when disrupted causes an illness (psychological dimension), which is the human experience of the disease (biological dimension) and that defines the subject as sick (social dimension )[4]. In the same line, normality as it is conceptualized in here also takes into account this subjective experience of disease, the social context of the individual and its biological aspects, integrating them in order to better understand the problem and to design an healthcare action plan. Denoting normality as a concept differentiated from health and disease, people are thus empowered to manage their health condition.

No longer should the individual consider himself/herself abnormal for having any health condition, but rather the emphasis of this concept of normality is for him/her to evaluate his/her normality in the attitude to manage it. This management can be made according to Ideal goals that work as reference points to the design of an action plan focused on the Process, which takes biostatistical information into account, and determines what is Health conforming to personal specificities, considering which can constitute Biological Advantages.

The current perspective over health and normality focuses autonomy, and autonomy surpasses other ethical values. In situations of less autonomy that before were regarded with a paternalistic look, where normality was a matter decided by experts, is now being replaced by a perspective that privileges the human being as the subject empowered to rule the decisions with impact in his/her health, in a smaller or larger scale. Accordingly, Human Rights are treasured and above all views on society conceptions, the individual being the center of everything, from social decisions to medical actions. An example of this is the European Council Recommendation four on principles concerning the legal protection of incapable adults, where it is stated that “there is a recognition that existing freedoms and capacities should be preserved as much as possible and those measures which needlessly take away people’s rights are indefensible”.

A model to normality must address diversity. And for this, the aspect of process is of great advantage. It is useful to consider that for normality to be achieved, what a person wants to achieve is important. Autonomy should be considered, and albeit possibly unhealthy, it is not abnormal. Individuals are entitled to transform themselves, both by changing their physical aspect and by practicing their values and preferences. The implications of using or refusing medical tools to enhance self-determination and what constitutes this must be socially discussed and legal frameworks created. An array of divisive topics could be named, such as abortion, euthanasia, transgender hormone therapy or drug abuse. However, while public opinion may judge with both legal and moral arguments, science must preserve its systematic and logical approach, observing and analyzing facts. In this way it favors the impartiality of the conception of what is normality in medicine and elevates discussions of other disciplines that are entitled to create boundaries. In a similar fashion of what could happen with normality in medicine, the entitlement to self-determination already protects individuals from public scrutiny in other domains as for example religious matters.

## Conclusion

The subject of normality in medicine underlies clinical practice and has several implications in public life, having been discussed since ancient times in those contexts.

Assuming the need for a clear concept and pursuing it is urgent and in this text the most accepted definitions in the academic community were analyzed and grouped into models. Then three case studies were chosen: anemia, psychiatric diseases and psychopathy, and commented in the light of these models.

Normality has been often synonym with health, either by the BST or other theories, and this is the most popular definition. Nevertheless, it has been employed meaning an ideal, a process or a biological advantage.

The conception of what is normal is not debated frequently and due to its philosophical character is hard to set clear questions and answers.

From this analysis the authors realize that the most common model that equates normality with health, although being intuitive is very infrequent, since there are very little individuals that are healthy strictly speaking and there are large communities of people highly adapted to living with diseases. Accordingly, these individuals would also be unfit for the other models of normality considered.

Therefore, the authors realize that these models require a redesign, and stress the importance of integrating aspects like variability and the respect for autonomy.

With the development in societies and advances in medical information and technologies a clear definition of its purposes demands further thinking about what is normality in medicine.

## Data Availability

Not applicable.
